# KRAS G12C inhibition enhances efficacy to conventional chemotherapy in KRAS-mutant NSCLC

**DOI:** 10.3389/fonc.2025.1654491

**Published:** 2025-09-10

**Authors:** Alessandro Tubita, Sara Fancelli, Lorenzo Anela, Giulia Petroni, Enrico Caliman, Francesca Mazzoni, Federico Scolari, Brunella Napolitano, Beatrice Menicacci, Camilla Eva Comin, Luca Voltolini, Serena Pillozzi, Lorenzo Antonuzzo

**Affiliations:** ^1^ Department of Experimental and Clinical Medicine, University of Florence, Florence, Italy; ^2^ Oncology Unit, Careggi University Hospital, Florence, Italy; ^3^ Department of Experimental and Clinical Biomedical Sciences, University of Florence, Florence, Italy; ^4^ Department of Health Sciences, University of Florence, Florence, Italy

**Keywords:** NSCLC, KRAS mutations, chemoresistance, KRAS inhibitors (KRASi), adjuvant chemotherapy

## Abstract

Despite recent therapeutic advances, the adjuvant treatment of non-small cell lung cancer (NSCLC) remains a challenge. Reducing the risk of recurrence is still a concern, especially in the KRAS G12C population, for which platinum-based adjuvant chemotherapy (CT) remains the gold standard. In this study, we evaluated the efficacy, in terms of cell viability and volumetric reduction, of adding KRAS inhibitors (KRASi) sequentially or concurrently to CT in both parental (PR) and gemcitabine-resistant (GR) KRAS mutated NSCLC cell lines (SW1573 and H23). We demonstrated that KRASi added to CT (both sequential and concurrent treatment strategies) reduced cell viability in SW1573-PR and H23-PR and this effect is less evident in GR cell lines. Interestingly, in the 3D model, the concomitant use of KRASi+CT reduced spheroid volume in both PR and GR spheroids. Our results indicate that KRASi enhances the efficacy of CT in both NSCLC PR and GR cells, suggesting a potential therapeutic strategy to overcome chemoresistance in the adjuvant setting of NSCLC.

## Introduction

Lung cancer is the leading cause of cancer-related mortality worldwide, with non-small cell lung cancer (NSCLC) accounting for approximately 85% of all lung cancer cases (1–3). The prognosis for NSCLC patients is often poor, even in early stages with a five-year survival rate between 26%-60% (4). 25% of NSCLC patients are diagnosed with an early-stage resectable disease, and for these patients surgery remains the primary therapeutic approach with curative intent. However, approximately 35-60% of these patients experience disease recurrence after surgery alone. Despite significant advancements in treatment modalities over the past decades, the management of post-operative NSCLC has been based on traditional chemotherapy (CT) platinum-based regimens with nucleoside analogs (e.g., gemcitabine) (5). Adjuvant CT provides only a 5.4% improvement in 5-year overall survival (OS) regardless of the choice of platinum-based treatments (6). The discovery of molecular alterations and oncogenic drivers in NSCLC has paved the way for targeted therapies, offering a new paradigm in cancer treatment. Recently, the integration of immunotherapy into the adjuvant setting for NSCLC with programmed death-ligand 1 (PD-L1) expression ≥50% (7), as well as targeted therapies for epidermal growth factor receptor (EGFR)-mutated disease (8) and anaplastic lymphoma kinase (ALK) fusions (9), has significantly improved survival outcomes in patients undergoing surgical treatment.

Currently, not all oncogene alterations known to have a therapeutic target in the metastatic setting have a treatment counterpart in earlier settings, including the adjuvant one. Among these, kirsten rat sarcoma viral oncogene homolog (KRAS) gene mutations are prevalent in approximately 30% of NSCLC cases and represent a critical therapeutic target (10–12). The majority of these mutations results in the replacement of glycine (G) in codon 12 with cysteine (C) (G12C), occurring in approximately 50% of KRAS mutant tumors. KRAS G12C mutations are strongly associated with tobacco exposure and KRAS G12C-mutant NSCLCs have been consistently reported to have a higher tumor mutational burden (TMB) and a high rate of concurrent mutations such as *STK11*, *KEAP1*, *SMARCA4* and *ATM* compared to NSCLCs carrying other KRAS isoforms or KRAS wild-type (WT) (13). However, the prognostic role of KRAS mutations is still unclear, although recent experience may suggest an unfavorable role compared to WT disease and when mutant KRAS NSCLC are associated with co-occurring mutations in advanced disease treated with chemo/chemoimmunotherapy (14, 15). The demonstrated efficacy of sotorasib and adagrasib, the first mutant-selective covalent KRAS G12C inhibitors (KRASi) in KRAS G12C pretreated NSCLC, with response rates of 30-40%, led to approval by the European Medicines Agency (EMA) and the Food and Drug Administration (FDA), marking a breakthrough for this drug category (16–19). However, there are currently no data on the efficacy of KRASi in the adjuvant setting, and the few available clinical trials are in early stages of enrollment (NAUTIKA-1, NCT04302025).

Given the poor efficacy of traditional adjuvant CT and the advent of KRAS-targeted therapies, there is a growing interest in exploring combination approaches with KRASi in early settings to enhance the therapeutic efficacy and overcome resistance mechanisms, thereby improving clinical outcomes for NSCLC patients. The present study investigates the potential of combining KRASi with standard chemotherapeutic agents in both parental (PR) and gemcitabine-resistant (GR) NSCLC cell lines. By harnessing 2D and 3D preclinical cellular models, we aim to elucidate the effects of these combinations, to determine whether the sequential or concomitant use of KRASi with CT modifies cell viability, and thus establish their potential for advancement in the therapeutic landscape of NSCLC.

## Materials and methods

### Patients

Patients with NSCLC who underwent surgery between 2019 and 2023 at the Clinical Oncology Unit of the Azienda Ospedaliero-Universitaria Careggi in Florence in Italy were enrolled. We collected data of patients stage II to IIIB per the Union Internationale Contre le Cancer and American Joint Committee on Cancer staging system (8th edition-2017) treated with adjuvant CT. We recorded demographic characteristics, type of surgery and adjuvant CT performed, stage, and biomolecular characteristics when available. Finally, we collected data on relapse-free survival (RFS) and overall survival (OS).

The study was conducted in accordance with good clinical practice (GCP) guidelines, the ethical principles of the Declaration of Helsinki, and regulatory requirements and local laws. The protocol was approved by the local ethics committee (CEAVC n.22712). All patients provided written informed consent.

### Adjuvant treatment and follow-up

Patients who were able to receive cisplatin-based CT underwent 4 cycles of cisplatin 75 mg/mq or carboplatin AUC5 IV D1 Q3W and gemcitabine 1250 mg/mq days IV D1,8 Q3W. Radiologic evaluation was performed according to the clinical practice schedule at baseline and then every 3 months with a whole body CT scan.

### Cell lines and culture

NSCLC cell lines (SW1573 and H23) with a KRAS G12C mutation were kindly provided by Dr. Azucena Esparís-Ogando (IBMCC-CIC, IBSAL, CIBERONC, Salamanca, Spain). NSCLC cell lines were cultured in Roswell Park Memorial Institute (RPMI)-1640 medium (Life technologies, Carlsbad, CA, USA) supplemented with 10% fetal bovine serum, L-glutamine (2mM), penicillin/streptomycin (50 U/ml) (Euroclone, Milan, Italy) at 37°C and 5% CO2. To generate GR-cells, SW1573 and H23 cells were transiently exposed to gemcitabine twice a week with increasing concentrations of gemcitabine weekly for more than 2 months.

### Drug treatments

The chemotherapeutic agents used in this study were carboplatin, gemcitabine, pemetrexed, and paclitaxel. The KRASi used were sotorasib and adagrasib. gemcitabine, sotorasib, adagrasib, pemetrexed and paclitaxel were purchased from MedChemExpress (Monmouth Junction, NJ, USA). carboplatin was provided by the Azienda Ospedaliera Universitaria Careggi’s galenic pharmacy (AOUC, Firenze, Italy).

### Cell viability assay

Cell viability was measured using Prestoblue™ Cell Viability reagent (Invitrogen, Waltham, MA, USA) according to the manufacturer’s protocol. The optical density (OD) was measured using a 560 nm excitation filter and 590 nm emission filter using the BioTek Synergy™ H1 hybrid multi-mode microplate reader (Agilent, CA, USA). Half-maximal inhibitory concentration (IC_50_) values were derived by a sigmoidal dose-response (variable slope) curve fitted using a four-parameter logistic regression model (log(inhibitor) vs. normalized response Variable slope (four parameters)) as described in the software documentation of Graph Pad Prism v6.0.

### Analysis of cell cycle

A total of 150–000 cells/well were seeded in 6-multiwell plates. After medium removal, 500 μl of solution containing 50 μg/mL propidium iodide, 0.1% w/v trisodium citrate and 0.1% NP40 was added. Samples were then incubated for 1 hour at 4°C in the dark and nuclei analyzed with FACSCanto flow cytometer (Becton Dickinson, Franklin Lakes, New Jersey, USA).

### Cell lysis and western blotting

Total cell lysates were obtained using Laemmli buffer (62.5 mM Tris-HCl-pH 6.8, 10% glycerol, 0.005% bromophenol blue, SDS 2%). Culture plates were placed on ice and cell monolayers were rapidly washed three times with ice-cold PBS containing 100 mM orthovanadate (Merck Millipore, Billerica, MA, USA). Cells were lysed by scraping in Laemmli buffer and incubating at 95°C for 10 min. Lysates were then clarified by centrifugation (13000 rpm for 10 min at room temperature). Proteins were separated on Bolt BisTris Plus gels 4–12% precast polyacrylamide gels (Life Technologies, Monza, Italy). Then, proteins were transferred from the gel to a polyvinylidene difluoride (PVDF) membrane using the iBlot 2 System (Thermo Fischer Scientific, Milan, Italy). Blots were blocked for 5 min, at room temperature, with the EveryBlot Blocking Buffer (BioRad, Hercules, CA, USA). Subsequently, the membrane was probed at 4°C overnight with primary antibodies diluted in a solution of 1:1 Immobilon^®^ Block–FL/T-PBS buffer (Merck Millipore, Billerica, MA, USA). The primary antibodies were as follows: Rabbit anti-p21Waf1/Cip1, rabbit anti-pRb-S807/811, mouse anti-Vinculin, rabbit anti-pERK1/2-T202/Y204, rabbit anti-pAKT-S473, rabbit anti-Beclin-1, rabbit anti-E-Cadherin, rabbit anti-Actin, mouse anti-p21, rabbit anti-cleaved-caspase 3, mouse anti-PCNA (1:1000, Cell Signaling Technology, Danvers, MA, USA), mouse anti-N-Cadherin (1:1000, DAKO Agilent, Milan, Italy), rabbit anti-CyclinB, mouse anti-CyclinD1 and mouse anti-Tubulin (1:1000, Santa Cruz Biotechnology, Santa Cruz, CA, USA). The membrane was washed in T-PBS buffer, incubated for 1 h with goat anti-rabbit IgG Alexa Fluor 750 antibody (1:30000) or with goat anti-mouse IgG Alexa Fluor 680 antibody (1:30000; Invitrogen, Monza, Italy), and then visualized at the Odyssey Infrared Imaging System (LI-COR Bioscience, Lincoln, NE, USA). Mouse anti-Vinculin or rabbit anti-Actin antibodies were used to assess an equal amount of protein loaded in each lane.

### Spheroid formation assay

SW1573-PR/GR and H23-PR/GR cells were seeded in RPMI 10% FBS in 96-well plate (2000 cells/well) precoated with 1.5% agarose (Condalab, Madrid, Spain) in water. After 72 hours, photos of time 0 were taken and spheroids were left untreated (CTRL) or treated with drugs. Photos were taken after 3 and 7 days of treatment by using Leica DM1 Inverted Microscope (Leica, Wetzlar, Germany) and the volume of SW1573-PR/GR or H23-PR/GR spheroids was quantified with ImageJ [Volume = 0.5*L*W2, L=length (major axis) W=width (minor axis)].

### RNA extraction and bulkRNAseq

Total RNA was extracted from NSCLC cell lines using the RNeasy Mini kit (Qiagen, Manchester, UK). The quantity and the quality of RNA were evaluated using a Nanodrop spectrophotometer (Thermo Fischer Scientific, Milan, Italy). RNA-seq was performed by Novogene using the Novaseq PE150 pipeline.

### Transcriptomic analysis

Raw sequencing data were assessed for quality. Reads were trimmed and aligned to the GRCh38 reference genome using HISAT2 (20). Alignment files were converted, sorted, and indexed using Samtools (21). Gene-level expression was quantified with featureCounts (22) and raw counts were loaded in an R environment. Differential expression analysis (DEA) was performed on raw counts using DESeq2 (23). Gene Set Enrichment Analysis (GSEA) was performed on the DEA results sorted by the Wald statistic using clusterProfiler (24). Pathways’ gene expression scores were calculated on normalized, log-scaled and variance-stabilized counts as the average expression of the pathway genes. P-values were corrected for multiple testing when necessary using the BH method.

### Statistical analysis

Cell viability and spheroid volumes are reported as mean ± SD of values obtained from at least three independent experiments. Clinical data are reported as absolute numbers and percentages. P values were calculated using the appropriate statistical test based on the distribution of the data and multiple testing corrections were applied when necessary using the Bonferroni method. Survival data were reported using the Kaplan-Meier estimator and comparison of survival times between groups were performed using a log-rank test.

## Results

### Population characteristics and survivorship

We identified a total of 47 patients with NSCLC who received adjuvant platinum- and gemcitabine-based CT (**Table 1**). 57.4% (n=27) of patients were aged 70 years or younger, and 74.5% (n=35) were male. Surgery consisted of lobectomy in 78.7% (n=37) and included lymphadenectomy in almost all cases (95.7%; n=45). Histological examination revealed that 91.5% (n=43) were adenocarcinomas, while the remaining 8.5% (n=4) were squamous cell carcinomas. The most common stages were IIB (57.4%; n=27) and IIIA (31.9%; n=15). 53.2% of patients had PD-L1 greater than or equal to 1 and 44.7% (n=21) had KRAS mutations. The most common KRAS mutations were G12C (42.9%; n=9), G12V (19%; n=4) and G12A (14.3%; n=3). 51.1% of patients (n=24) experienced a recurrence, which involved lung in 50% of cases (n=12), lymph nodes in 33.3% (n=8), and the central nervous system (CNS) in 16.7% (n=4).

**Table 1:** Patient clinical, and molecular characteristics stratified by KRAS mutation status.

		TOT	KRAS		P-value
		N=47	mut (n=21)	wt (n=26)	
Age	<70	27 (57.4%)	12 (44.4%)	15 (55.6%)	
	≥70	20 (42.6%)	9 (45%)	11 (55%)	1
Gender	F	12 (25.5%)	2 (16.6%)	10 (83.4%)	
	M	35 (74.5%)	19 (54.3%)	16 (45.7%)	0.042
Lymphadenectomy	NO	2 (4.3%)	2 (100%)	0 (0%)	
	YES	45 (95.7%)	19 (42.2%)	26 (57.8%)	0.194
Resection type	Lobectomy	37 (78.7%)	13 (35.1%)	24 (64.9%)	
	Pyramidotomy	1 (2.1%)	1 (100%)	0 (0%)	
	Pneumonectomy	2 (4.3%)	2 (100%)	0 (0%)	
	Atypical resection	3 (6.4%)	2 (66.6%)	1 (33.4%)	
	Segmentectomy	4 (8.5%)	3 (75%)	1 (25%)	0.071
Histology	Squamous cell carcinoma	4 (8.5%)	1 (25%)	3 (75%)	
	Adenocarcinoma	43 (91.5%)	20 (46.5%)	23 (53.5%)	0.617
pT	pT1	9 (19.1%)	2 (22.2%)	7 (77.8%)	
	pT2	14 (29.8%)	6 (42.8%)	8 (57.2%)	
	pT3	19 (40.4%)	10 (47.6%)	9 (52.4%)	
	pT4	5 (10.6%)	3 (60%)	2 (40%)	0.443
pN	pN0	17 (36.2%)	10 (58.8%)	7 (41.2%)	
	pN1	15 (31.9%)	3 (20%)	12 (80%)	
	pN2	13 (27.7%)	6 (46.1%)	7 (53.9%)	
	pNx	2 (4.3%)	2 (100%)	0 (0%)	0.054
pM	pM0	47 (100%)	21 (44.7%)	26 (55.3%)	
Stage	IIA	1 (2.1%)	1 (100%)	0 (0%)	
	IIB	27 (57.4%)	11 (40.7%)	16 (59.3%)	
	IIIA	15 (31.9%)	7 (46.7%)	8 (53.3%)	
	IIIB	4 (8.5%)	2 (50%)	2 (50%)	0.806
PD-L1	<1%	22 (46.8%)	7 (33.3%)	15 (57.7%)	
	≥1%	25 (53.2%)	14 (66.7%)	11 (42.3%)	0.171
KRAS status	mut	21 (44.7%)	21 (100%)	0 (0%)	
	wt	26 (55.3%)	0 (0%)	26 (100%)	
Adjuvant therapy	plat + gem	47 (100%)	21 (44.7%)	26 (55.3%)	
First line therapy	NO	23 (48.9%)	9 (39.1%)	14 (60.9%)	
	YES	24 (51.1%)	12 (50%)	12 (50%)	0.649
Relapse site	Distance	10 (41.7%)	5 (50%)	5 (50%)	
	Local	13 (54.2%)	7 (53.9%)	6 (46.1%)	
	Both	1 (4.2%)	0 (0%)	1 (100%)	
	NA	23	9	14	1
Metastasis CNS	NO	20 (83.3%)	11 (55%)	9 (45%)	
	YES	4 (16.7%)	1 (25%)	3 (75%)	
	NA	23	9	14	0.59
Nodal metastasis	NO	16 (66.7%)	8 (50%)	8 (50%)	
	YES	8 (33.3%)	4 (50%)	4 (50%)	
	NA	23	9	14	1
Lung metastasis	NO	12 (50%)	4 (33.3%)	8 (66.7%)	
	YES	12 (50%)	8 (66.7%)	4 (33.3%)	
	NA	23	9	14	0.22
Bone metastasis	NO	21 (87.5%)	10 (47.6%)	11 (52.4%)	
	YES	3 (12.5%)	2 (66.7%)	1 (33.3%)	
	NA	23	9	14	1
Other metastasis	NO	18 (75%)	10 (55.5%)	8 (45.5%)	
	YES	6 (25%)	2 (33.3%)	4 (66.7%)	
	NA	23	9	14	0.64
KRAS mutation types	G12A	3 (14.3%)			
	G12C	9 (42.9%)			
	G12D	1 (4.8%)			
	G12S	1 (4.8%)			
	G12V	4 (19%)			
	Q61L	1 (4.8%)			
	UNK	2 (9.5%)			

The table summarizes the distribution of age, gender, surgical procedures, histological subtypes, tumor staging (pT, pN, pM, and overall stage), PD-L1 expression, and KRAS mutation details. Statistical analyses, including p-values by Fisher’s Exact Test, are provided to indicate significant differences between KRAS-mut and KRAS-wt groups. Additional columns report treatment modalities, metastasis locations, and KRAS mutation types. Abbreviations: CNS, Central nervous system; UNK, Unknown.

The bold values were those significant in the table.

Comparing the two subgroups of KRAS mutated and KRAS WT patients for demographic and disease characteristics, we found that WT patients are more likely to be female (83.4%; n=10, Fisher’s Exact Test p=0.042), stage IIB (59.3%; n=16) or IIIA (53.4%; n=8, p=0.806), and are PD-L1 <1% (68.2%; n=15, p=0.171). In contrast, the KRAS mutated subgroup has a similar distribution of male and stage of disease presentation, but is more likely to be PD-L1 ≥1% (56%; n=14). Moreover, although not statistically significant (p=0.054), WT patients are more likely to have positive lymph nodes pN1 (80%;n=12) and pN2 (53.9%; n=7) than mutated patients pN1 (20%; n=3) and pN2 (46.1%; n=6). Finally, both groups have the same probability of recurrence with a higher incidence of CNS metastases in the WT subgroup (75%; n=3) (p=0,59).

**Figure 1:**
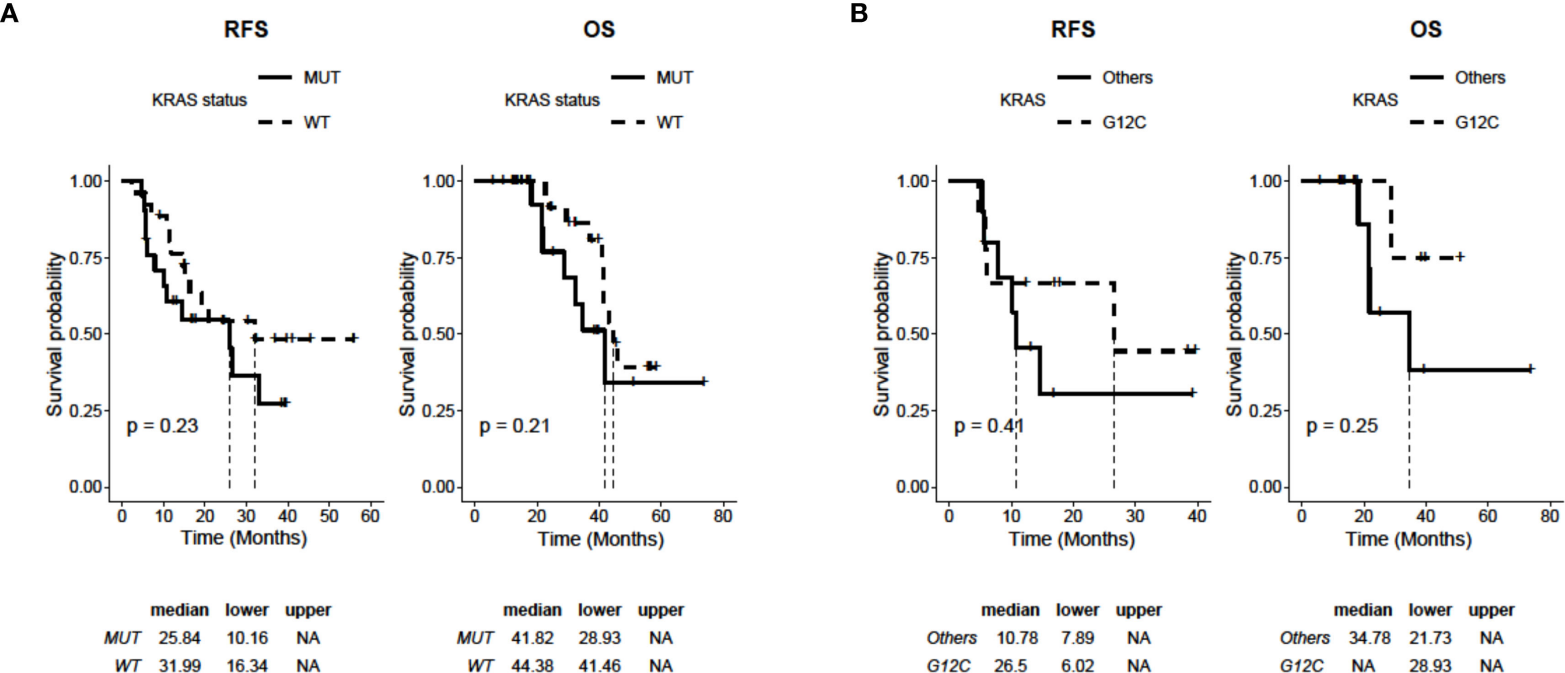
Kaplan-Meier analysis of RFS and OS depending on the status of KRAS in 47 NSCLC patients (WT: n=26; mutated: n=21). **(A)** RFS and OS curves of patients with WT and mutated KRAS (KRAS mutations: G12C, G12A, G12D, G12S, G12V, Q61L). **(B)** RFS and OS curves of patients with KRASG12C mutation (n=9) and KRAS mutated in other isoforms (i.e., G12A, G12D, G12S, G12V, Q61L; n=12). P values were calculated with a log-rank test.

To define the predictive and prognostic value of KRAS mutations, we compared the mutated and WT patient populations of our cohort for RFS and OS. Although they did not reach statistical significance, we observed a slight trend in favor of the WT subgroup with median RFS (mRFS) of 31.99 months (95% CI: 16.34-NA) compared to 25.84 months (95% CI: 10.16-NA) of the mutated group (*p=0.23*). Similarly, OS also tends to favor the WT population over the mutated population (mOS 44.38 months 95% CI: 28.93-NA vs. 41.82 months 95% CI: 41.46-NA, *p=0.21*) (**Figure 1A**). Finally, within the subgroup of patients with KRAS mutations, we evaluated the RFS and OS of G12C compared to the other mutations, showing a non-statistically significant advantage for both endpoints. In particular, mRFS was 26.5 months (95% CI: 6.02-NA) for G12C compared to 10.78 months (95% CI: 10.78-NA) for the others (*p=0.47*). For the G12C mutation, mOS was not reached (95% CI: 28.93-NA), while it was 34.78 months (95% CI: 21.73-NA) for the remaining mutations (p=0.25; **Figure 1B**).

### Enhanced efficacy of carboplatin and gemcitabine combined with KRASi in reducing viability in KRAS-mutated NSCLC cell lines

**Figure 2:**
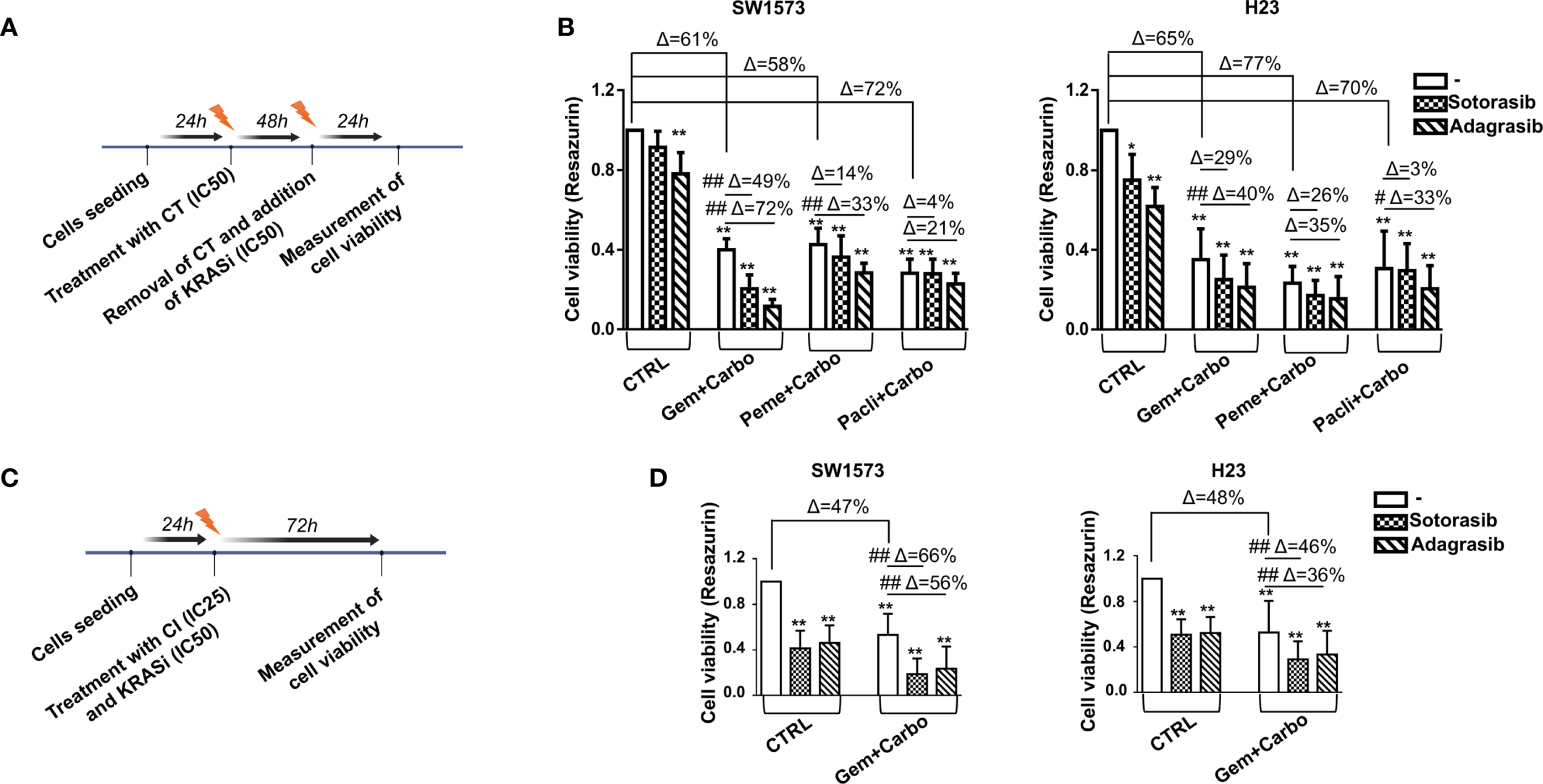
Chemotherapeutic agents sensitized NSCLC cells to KRASi in 2D cell viability assay. **(A)** The cartoon indicates the sequential treatment schedule used for the cell viability assay. Initially the cells were seeded and after 24 hours they were treated with chemotherapeutic (CT) agents for 48 hours at their IC_50_. After this amount of time, these agents were removed and a KRAS inhibitor was administered for additional 24 hours at their IC_50_. **(B)** Cell viability assay was performed in SW1573 or H23 cells treated with combination of chemotherapeutic agents (gemcitabine+carboplatin or pemetrexed+carboplatin or paclitaxel+carboplatin) and KRASi (sotorasib or adagrasib) following the time schedule reported in **(A)**. Data shown are mean ± SD from three independent experiments. *P < 0.05; **P < 0.01 refer to differences vs untreated cells (CTRL) as determined by Student t test; #P < 0.05; ##P < 0.01 refer to differences between the indicated samples as determined using one-way ANOVA. Δ indicates the percentage change in cell viability relative to CTRL cells or between the indicated samples. **(C)** The cartoon indicated the concomitant treatment schedule used for the cell viability assay. Initially the cells were seeded and after 24 hours they were treated with a combination of Gem+Carbo (CI) at their IC_25_ and KRASi at their IC_50_ for 72 hours. **(D)** Cell viability assay was performed in SW1573 or H23 cells treated with combination of chemotherapeutic agents Gem+Carbo and KRASi adagrasib following the schedule reported in **(C)** Data shown are mean ± SD from three independent experiments. **P < 0.01 refer to differences with respect to control (CTRL) as determined by Student t test; ##P < 0.01 refer to differences between the indicated samples as determined using one-way ANOVA. Δ indicates the percentage change in cell viability relative to the control (CTRL) or between the indicated samples.

To define the optimal combination of chemotherapeutic agents, among those commonly used in NSCLC in adjuvant settings, that are capable of maximizing sensitivity to KRASi treatment, we developed two experimental protocols: one based on sequential treatments and the other based on concurrent treatments. Chemosensitivity was quantified as the IC_50_ value, representing the drug concentration required to achieve a 50% reduction in cell viability. The IC_50_ values for chemotherapeutic agents and KRASi (sotorasib or adagrasib) were derived from dose-response curves (**Table 2**; **Supplementary Figure S1**). Of note, SW1573 cell line has been reported to be more resistant to sotorasib compared to the H23 cell line (25). In general, with the exception of gemcitabine, our experiments demonstrate that the SW1573 cells exhibit significantly higher IC_50_ values for all other drugs tested, highlighting their increased resistance profile. Using the sequential treatment scheme (**Figure 2A**), KRAS-G12C-mutated NSCLC cell lines (SW1573 and H23) were seeded and after 24 hours were treated with a combination of chemotherapeutics, including gemcitabine (IC50 H23: 3.3nM-SW1573: 4nM) plus carboplatin (IC50 H23: 30μM-SW1573: 64μM) (Gem+Carbo), pemetrexed (IC50 H23: 2.3nM-SW1573: 37μM) plus carboplatin (Peme+Carbo), and paclitaxel (IC50 H23: 134μM-SW1573: 245μM) plus carboplatin (Pacli+Carbo), for 48 hours at their IC_50_. Control cells (CTRL) were maintained without any chemotherapeutic agents or KRASi for 72 hours. After 48 hours of treatment, the chemotherapeutics were removed, and the cells were subsequently exposed to KRASi (sotorasib or adagrasib; IC50 sotorasib H23: 540nM-SW1573: 65μM; IC50 adagrasib H23: 200nM-SW1573: 4μM) for an additional 24 hours at their IC_50_.

**Table 2:** IC_50_ values for chemotherapeutic agents and KRAS inhibitors in NSCLC cell lines.

	SW1573 (µM)	H23 (µM)
Carboplatin	64h4.4	30 ± 6
Gemcitabine	0.004 ± 0.0006	0.0033 ± 0.00025
Sotorasib	65 ± 4.9	0.540 ± 0.014
Adagrasib	4 ± 0.5	0.200 ± 0.015
Pemetrexed	37 ± 0.6	0.0023 ± 0.003
Paclitaxel	245 ± 7.2	134 ± 0.021

The results were expressed as the percentage change in cell viability (Δ%) relative to the control or between the indicated samples. Significant differences were observed in cell viability across different treatment groups, with variations between the responses of SW1573 and H23 cell lines. Importantly, although the Gem+Carbo condition did not achieve the highest percentage of growth inhibition among all tested chemotherapeutic combinations as compared to untreated cells (SW1573: Gem+Carbo Δ=61% vs Peme+Carbo Δ=58% and Pacli+Carbo Δ=72%; H23: Gem+Carbo Δ=65% vs Peme+Carbo Δ=58% and Pacli+Carbo Δ=72%), it proved to be the most effective combination respect to the other combined treatments in sensitizing SW1573 and H23 cells to sotorasib (Δ=49% and Δ=29%, respectively compared to Gem+Carbo-treated cells) and adagrasib (Δ=72% and Δ=40%, respectively compared to Gem+Carbo-treated cells) (**Figure 2B**).

For the experimental design in which treatments were administered concurrently, firstly we tested the best combination of chemotherapeutic agents (Gem+Carbo) and KRASi at the IC_25_ or at the IC_50_ concentrations for 72 hours in SW1573 and H23 cell lines (**Supplementary Figure S2**). Importantly, using this experimental design at the IC_25_ concentrations, for the Gem+Carbo treatment we achieved a similar reduction in cell viability, comparable to the sequential treatment scheme, at least in the H23 cell line. Specifically, in H23 cells the viability reduction due to gem+carbo combined treatment was substantial (Δ = 65%), while in SW1573 cells, the decrease was more moderate (Δ = 46%). A higher decrease was obtained when the chemotherapeutic agents and KRASi were administered together at the IC_50_, and with a smaller, but significant, reduction when the drugs were administered at the IC_25_ for 72 hours in both cell lines (**Supplementary Figure S2**).

In view of these results, we decided to use the best chemotherapeutic combination Gem+Carbo at the IC_25_ combined with KRASi at the IC_50_, for 72 hours in both cell lines (**Figure 2C**) since we obtained similar effects when the chemotherapeutic agents and KRASi were administered together (SW1573: Gem+Carbo+Soto Δ=82% and Gem+Carbo+Ada Δ=77%; H23: Gem+Carbo+Soto Δ=72% and Gem+Carbo+Ada Δ=67%), while reducing the toxicity of chemotherapeutic agents. As expected, the administration of Gem+Carbo (this combination will be indicated as “CI” from here on) induced a slighter reduction of cell viability compared to the previous experiment (SW1573: CI Δ=47%; H23: CI Δ=48%), but determined a robust decrease of cell viability when used in combination with KRASi in SW1573 and H23 NSCLC cell lines (SW1573: CI+sotorasib Δ=66%, CI+adagrasib Δ=56%; H23: CI+sotorasib Δ=46% CI+adagrasib Δ=36%; compared to CI alone. **Figure 2D**). Thus, the combination of Gem+Carbo effectively enhances the sensitivity of KRAS-G12C-mutated NSCLC cell lines to KRASi, with both sequential and concurrent treatment strategies, achieving significant reductions in cell viability.

### Effect of chemotherapeutic agents and KRASi on spheroid volume reduction in parental KRAS mutated cell lines

**Figure 3:**
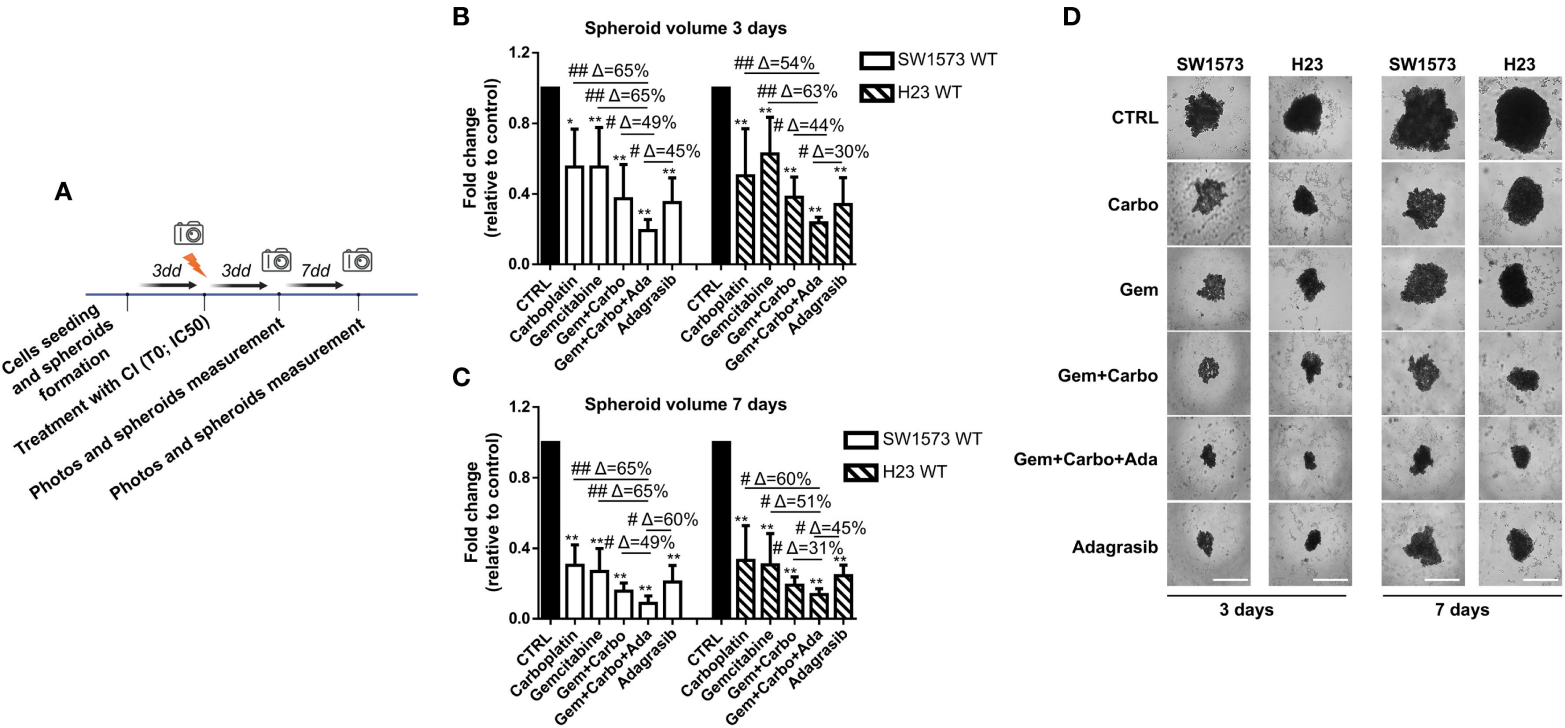
Chemotherapeutic agents sensitized NSCLC cells to KRASi adagrasib in a 3D spheroids model. **(A)** The cartoon indicated the treatment schedule used for the spheroid assay. Initially the cells were seeded and we waited 72 hours for spheroid formation. Then after this time, photos of time 0 were taken and spheroids were treated with chemotherapeutic agents or KRASi at their IC50. After that, photos were taken also after 3 and 7 days and spheroid volume was quantified each time. **(B)** SW1573 or H23 spheroids were treated with different combinations of chemotherapeutic agents and KRASi adagrasib at their IC_50_ for 3 days. **(C)** SW1573 or H23 spheroids were treated with different combinations of chemotherapeutic agents and KRASi adagrasib at their IC_50_ for 7 days. Graphs **(A, B)** show the quantification of spheroid volumes ± SD at different time points (3 and 7 days) normalized for the time point 0 (n = 3 independent experiments). **(D)** Representative images of spheroids taken at day 3 and 7 are shown. *P < 0.05; **P < 0.01 refer to differences with respect to control (CTRL) as determined by Student t test; #P < 0.05; ##P < 0.01 refer to differences between the indicated samples as determined using one-way ANOVA. Scale bar: 400 μm.

Three-dimensional tumor spheroids grown *in vitro* are extensively utilized as 3D cell culture models for anticancer drug evaluation because they closely mimic the physiological conditions of tumor tissue compared to 2D models (26). SW1573 and H23 spheroids were generated following this procedure: firstly cells were seeded to allow the formation of spheroids and after 72 hours, photos of time 0 (T0) were taken and spheroids were then treated with drugs or left untreated (CTRL). Photos were taken after 3 and 7 days of treatment and the volume of SW1573 or H23 spheroids was quantified (**Figure 3A**). We used SW1573 and H23 spheroids to test whether using the combination of CI and the KRASi adagrasib, which has demonstrated greater efficiency in reducing cell viability compared to sotorasib (**Figure 2**), at the IC_50_ concentrations leads to a similar response with respect to 2D models. At 3 days, the combination of CI and adagrasib showed a reduction between 45% and 65% in SW1573 spheroid volumes and a reduction between 30% and 63% in H23 spheroid volumes compared to the respective single-agent treatments or CI (**Figure 3B**). At 7 days, the effect was maintained (**Figures 3C, D**).

These findings confirm the data obtained from the 2D assay, and further demonstrate that the CI+adagrasib combination exhibits superior efficacy even when the cells are cultured in a 3D model.

### Generation and characterization of gemcitabine resistant cells

**Figure 4:**
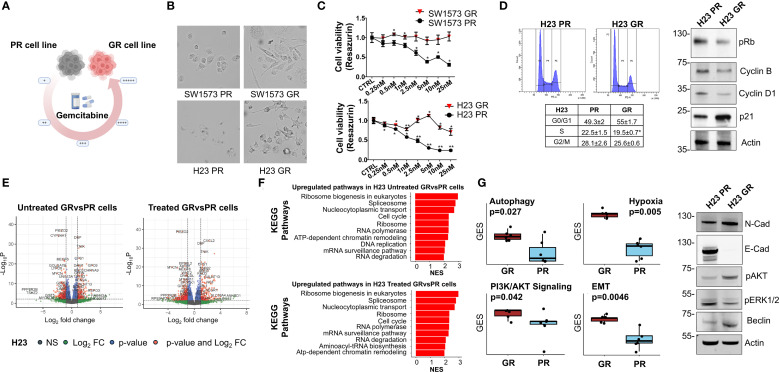
Characterization of gemcitabine resistance NSCLC cells **(A)** Schematic representing the generation of gemcitabine-resistant (GR) NSCLC cells from parental NSCLC cells, using incrementally increasing concentrations of gemcitabine in culture over time. **(B)** Representative images of PR and GR NSCLC cells. Cells were grown to 50% confluency and then photographed under 10× magnification. **(C)** Determination of cell viability for gemcitabine in PR and GR SW1573 and H23 NSCLC cells. Cells were treated with gemcitabine at the indicated concentrations for 72 h and viability was determined. Data were normalized to control and presented as mean ± SD from three independent experiments. *P < 0.05; **P < 0.01 refers to differences with respect to control (CTRL) as determined by Student t test. **(D)** Cell cycle phase distribution plots and values (tables) of GR NSCLC cells and of PR NSCLC cells (H23). Cells were analyzed after 48 h from cell seeding. Data shown are mean ± SD from three independent experiments. *p < 0.05 as determined by Student’s t-test (left). Immunoblot showing the expression or phosphorylation status of cell cycle regulators in H23-PR and H23-GR cells cultured for 48h (right). Actin was used as a loading control. **(E)** Volcano plot of differentially expressed genes (DEG) between H23-PR and H23-GR cells. H23-PR and H23-GR cells were treated with gemcitabine for 72 hours. Data are from three independent experiments. **(F)** KEGG pathways Gene Set Enrichment analysis of the DEGs between H23-PR and H23-GR cells. Only the ten most significantly upregulated pathways in H23-GR cells are shown. NES: Normalized Enrichment Score. **(G)** Differences in the expression of gemcitabine resistance-associated pathways in H23-PR and H23-GR cells (left). GES: Gene Expression Score. P-values have been computed with a Wilcoxon rank-sum test. Immunoblot showing the expression or phosphorylation status of resistance markers in H23-PR and H23-GR cells cultured for 72h (right). Actin was used as loading control.

Our experiments showed that gemcitabine is the most promising platinum partner in SW1573 and H23 cell lines inhibition, especially in sensitizing NSCLC cells to treatment with KRASi (**Figure 2B**). Notably, tumor cells often develop multidrug resistance after CT; therefore, to investigate the impact of acquired resistance to gemcitabine to the efficacy of KRASi, we generated NSCLC cell lines (SW1573 and H23) resistant to gemcitabine by chronic and repeated exposure to increasing gemcitabine concentrations (**Figure 4A**). We observed evident morphological differences between parental (PR) and resistant (GR) NSCLC cells. The SW1573-GR cells acquired a long spindle shape compared to the round shape of the PR cells. Furthermore, the SW1573-GR show a larger volume compared to PR cells. The H23-GR cells have developed pseudopodia and these are also larger than the PR counterpart (**Figure 4B**). GR cells were validated using cell viability assay, comparing PR and GR cell proliferation after a 72h of gemcitabine treatment. Compared to parental cells, H23-GR and SW1573-GR cells showed a slight decrease in cell viability upon treatment with increasing doses of gemcitabine (**Figure 4C**). To investigate the impact of gemcitabine resistance on cell proliferation, we performed a cell cycle analysis. In the H23-GR cell line, we observed a slight increase in the proportion of cells in the G0/G1 phase, and consequently a reduction in the S and G2/M phases, with respect to H23-PR. Consistently, in the H23-GR cell line we observed reduced levels of pRB, cyclin D1 and B1. We also found an increased expression of the cyclin-dependent kinase inhibitor (CDKi) p21, confirming the slight slowdown of the cell cycle in the gemcitabine-resistant cell line (**Figure 4D**). Then, we performed a comprehensive analysis of differentially expressed genes (DEGs) and KEGG pathway enrichment in H23 cell lines, including both PR and GR variants, under two conditions: untreated and treated with gemcitabine. This dual comparison enabled us to identify specific genes and pathways associated with the development and maintenance of gemcitabine resistance. By analyzing RNA-seq data from H23-PR and -GR cells in untreated and treated conditions, we identified 166 upregulated and 268 downregulated genes in untreated GR vs. PR cells, while 196 upregulated and 326 downregulated genes were observed in treated GR vs. PR cells (|log2FC| > 1; p-value < 0.05). These DEGs are visualized in volcano plots (**Figure 4E**). The top 10 genes that were consistently up-regulated in both untreated and treated GR cells were SLC4A4, TNFSF15, IGFBP3, ZNF711, CD36, CHRNA9, PLXDC2, GALNT13, PLCH1, UBE2QL1. While the top 10 genes that were consistently down-regulated in both untreated and treated GR cells were MYCN, SFRP5, DOK5, CRTAC1, TRPA1, CSMD2, GOLGA7B, RGS6, CYP24A1, TMEM179. To explore the functional implications of these transcriptional changes, we conducted a KEGG pathway analysis, identifying key pathways involved in mechanisms driving or contributing to drug resistance in both treated and untreated conditions (**Figure 4F**). In both conditions, the most enriched pathways are largely centered around protein synthesis (ribosome biogenesis, ribosome, RNA polymerase), RNA processing (spliceosome, mRNA surveillance), DNA metabolism (DNA replication, chromatin remodeling) and cell cycle regulation (**Figure 4F**). This upregulation shifts towards enhanced transcriptional and translational machinery, which may support an adaptation of resistant cells to survive DNA damage caused by gemcitabine. To delve deeper and to identify the main pathways involved in the mechanism of resistance to gemcitabine, we performed a gene set enrichment analysis on Gene Ontology pathways comparing H23-PR- and H23-GR-cells. This allowed us to identify multiple cellular pathways associated with gemcitabine resistance, including autophagy, PI3K/AKT signaling, epithelial-mesenchymal transition (EMT), and hypoxia response (**Figure 4G**). To confirm these findings at protein level, we performed Western blot analysis. In the H23-GR cell line, we observed a decrease in E-cadherin expression and an increase in N-cadherin expression, a common characteristic of EMT. Additionally, this resistant cell line exhibited an increase in Beclin-1, a marker of autophagy, as well as enhanced AKT phosphorylation, accompanied by a decrease in ERK1/2 phosphorylation (**Figure 4G**). Moreover, we observed that combined treatments of CI and CI+adagrasib result in modulation of the pERK1/2 pathway. Regarding AKT phosphorylation, which is more active in the gemcitabine-resistant cell line, was not modulated by the combined treatments (**Supplementary Figure S3A**). Given all of that, these findings suggest that gemcitabine resistance is associated with adaptive mechanisms that promote cell survival and therapy resistance.

### Effect of chemotherapeutic agents and adagrasib on GR NSCLC cells

**Figure 5:**
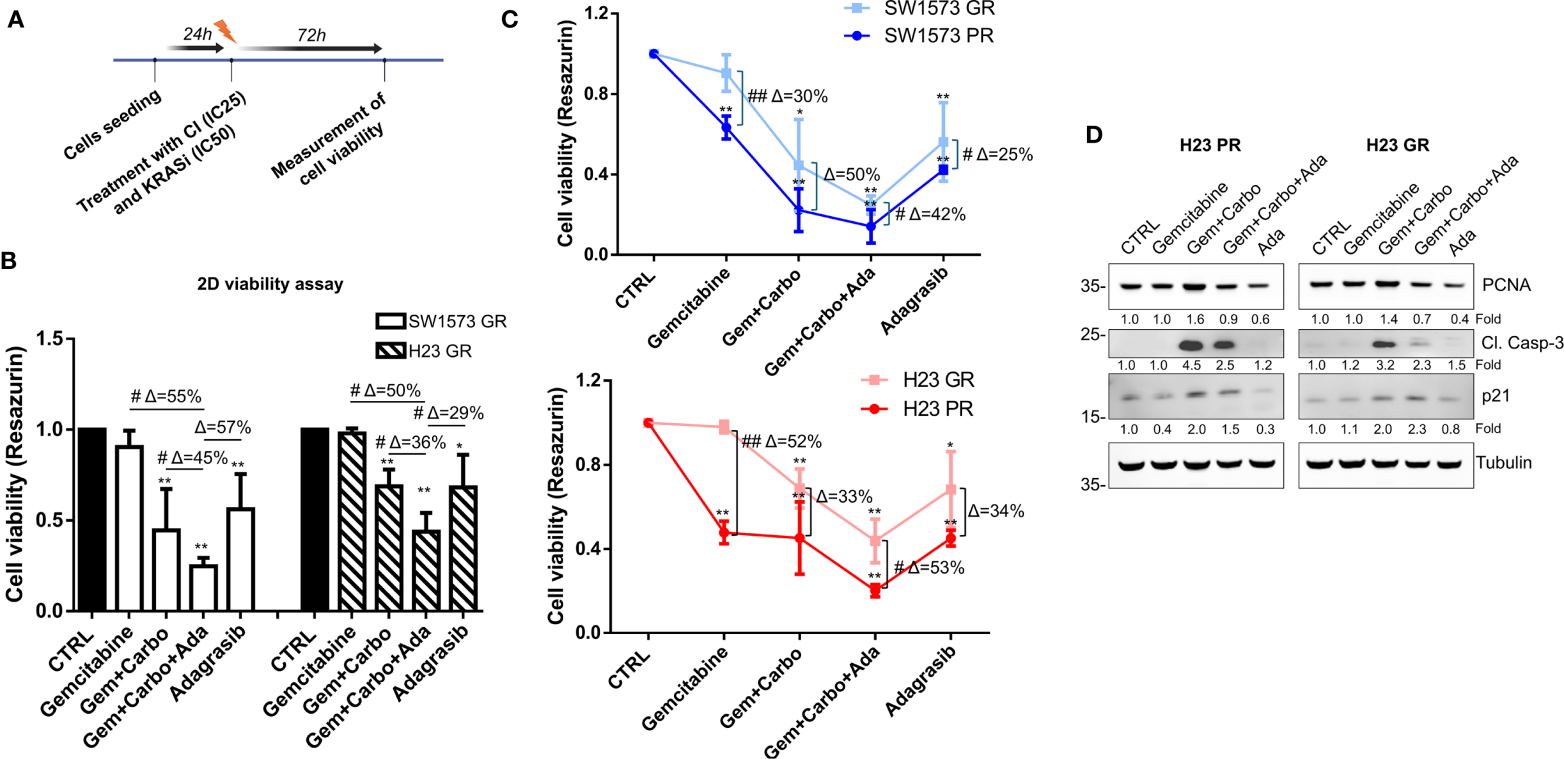
Chemotherapeutic agents sensitized gemcitabine resistant NSCLC cells to KRASi adagrasib in 2D cell viability assay. **(A)** The cartoon indicated the treatment schedule used for the cell viability assay. Initially the cells were seeded and after 24 hours they were treated with a combination of chemotherapeutic agents at their IC_25_ and KRASi at their IC_50_ for 72 hours. **(B)** Cell viability assay was performed in SW1573 or H23 cells treated with combination of chemotherapeutic agents gemcitabine and carboplatin and KRASi adagrasib following the time schedule of **Figure 2C**. Data shown are mean ± SD from three independent experiments. *P < 0.05; **P < 0.01 refer to differences with respect to control (CTRL) as determined by Student t test; #P < 0.05; ##P < 0.01 refer to differences between the indicated samples as determined using one-way ANOVA. Δ indicates the percentage change in cell viability relative to the control (CTRL) or between the indicated samples. **(C)** Determination of cell viability for gemcitabine comparing parental and GR SW1573 and H23 NSCLC cells. Cells were treated as indicated in **Figure 4B**. Data were normalized to control and presented as mean ± SD from three independent experiments. *P < 0.05; **P < 0.01 refers to differences with respect to control (CTRL) as determined by Student t test. #P < 0.05; ##P < 0.01 refer to differences between the indicated samples as determined using one-way ANOVA. **(D)** Expression of cleaved-caspase 3, PCNA and p21 in H23-PR and H23-GR cell line treated with the indicated drug combination for 72h detected by western blotting. Tubulin was used as loading control.

Next, we evaluated whether NSCLC cells, with different gemcitabine sensitivities (PR and GR), exhibit a different response to KRASi adagrasib. Firstly, we used SW1573-GR and H23-GR to assess cell viability after a 72 h treatment using CI at IC_25_ and KRASi adagrasib at IC_50_ (values determined in parental cell lines, see **Table 2**), following the same experimental schedule of **Figure 2C** (**Figure 5A**). While both GR cell lines showed resistance to gemcitabine as expected, the combination of CI+adagrasib was more effective respect to CI treatment alone and more significant accentuated in SW1573-GR (*Δ*=45%) compared to H23-GR (*Δ*=36%; **Figure 5B**). Importantly we observed a significant reduction in cell viability of PR and GR cell lines with the combination of CI and adagrasib although this effect is less evident in GR cell lines (SW1573GR vs PR: Gem+Carbo+Ada Δ=42%; H23GR vs PR: Gem+Carbo+Ada Δ=53%) (**Figure 5C**). To investigate the mechanisms underlying the reduction in cell viability, we observed a modulation of the Cleaved-Caspase-3 in the PR cell line following treatment with Gem+Carbo, as well as with the triple combination of CI+KRAS inhibitor. Interestingly, a similar modulation was also detected in the GR cell line, although to a lesser extent, suggesting a differential apoptotic response between the two models. We also evaluated the expression of additional proliferation- and cell cycle-related markers, including PCNA and p21. We observed a reduction in PCNA signal following treatment with Adagrasib alone and in combination with Gem+Carbo in both cell lines, indicating decreased proliferative activity. Additionally, p21 expression was upregulated upon treatment with Gem+Carbo, both in the presence and absence of the KRAS inhibitor, indicating activation of cell cycle arrest mechanisms. Notably, the increase was more pronounced in GR cell line compared to their parental counterparts, especially with CI+Adagrasib combination treatment, as confirmed by densitometric analysis (**Figure 5D**). These findings support the impact of the treatments on both apoptotic and proliferative pathways.

### Effect of chemotherapeutic agents and adagrasib on spheroid volume reduction of GR NSCLC cell lines

**Figure 6:**
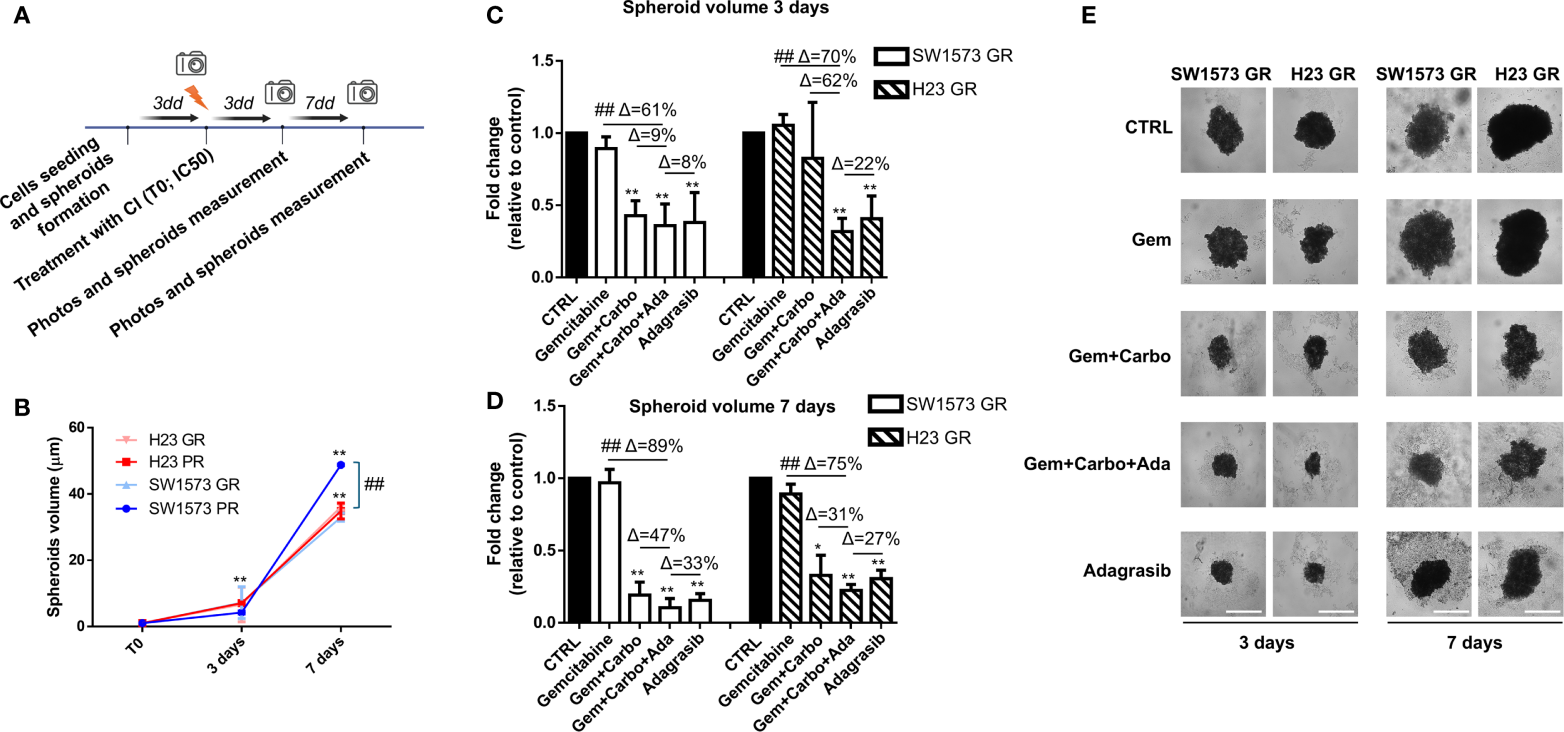
Chemotherapeutic agents sensitized gemcitabine resistant NSCLC cells to KRASi adagrasib in a 3D spheroids model. **(A)** The cartoon indicated the treatment schedule used for the spheroid assay. Initially the cells were seeded and spheroids were allowed to form for 72 hours. After this time, photos of time 0 were taken and spheroids were treated with chemotherapeutic agents or KRASi at their IC50. Photos were then taken after 3 and 7 days and spheroid volume was quantified each time. **(B)** Quantification of spheroid volumes ± SD at different time points (3 and 7 days) normalized for the time point 0 (n = 3) in PR and GR NSCLC cell lines. *P < 0.05; **P < 0.01 refers to differences with respect to control (CTRL) as determined by Student t test. ##, P < 0.01 refers to the difference between SW1573 GR and SW1573 PR as determined by Student t test. **(C)** SW1573 or H23 spheroids were treated with different combinations of chemotherapeutic agents and KRASi adagrasib at their IC_50_ for 3 days **(D)** GR SW1573 or H23 spheroids were treated with different combinations of chemotherapeutic agents and KRASi adagrasib at their IC_50_ for 7 days. Graphs **(A, B)** show the quantification of spheroid volumes ± SD at different time points (3 and 7 days) normalized for the time point 0 (n = 3). **(E)** Representative images of spheroids taken at day 3 and 7 are shown. *P < 0.05; **P < 0.01 refers to differences with respect to control (CTRL) as determined by Student t test. ##P < 0.01 refer to differences between the indicated samples as determined using one-way ANOVA. Scale bar: 400 μm.

Then, we studied the effect of combination therapies with CI and adagrasib to inhibit viability in GR NSCLC tumor spheroids using the same experimental schedule of **Figure 3A** (**Figure 6A**). Importantly, the spheroids generated from H23 PR and GR cells exhibit the same volume at all time points, whereas the spheroids derived from SW1573 PR and GR cells show a significant difference at 7 days (**Figure 6B**). As shown in **Figures 6C, D**, we observed a substantial and significant reduction in spheroid volume with different treatments in both cell lines (**Figures 6C, D**). At 3 days, the combination of CI and adagrasib led to reductions in spheroid volumes ranging from 8% to 61% for SW1573 and from 22% to 70% for H23 when compared to the corresponding single-agent treatments or CI alone (**Supplementary Figure S3B**, **Figure 6C**). By 7 days, these effects became more pronounced, with the SW1573 cell line showing a marked response to combination therapy, achieving up to an 89% reduction in spheroid volume, while the H23 cell line demonstrated a maximum reduction of 75% under similar conditions (**Figure 6D**).

Finally, at 7 days in GR spheroids the percentages of inhibition (*Δ*) of the combined treatment CI+adagrasib were the same as the parental type 3D models and the reduction of the volumes were similar with respect to parental cells (**Supplementary Figure S3C**). These findings are crucial as they provide evidence that the CI+adagrasib combination is effective also in resistant cells.

## Discussion

Adjuvant CT in NSCLC has a limited impact on survival, and for decades has been the only treatment available for stage II and III patients undergoing radical surgery. This lack of benefit is notable for EGFR mutations and ALK fusions, for which it has recently introduced targeted agents into clinical practice with significant survival benefits over CT alone (8, 9). However, with the exception of the mutations listed above, therapies for other known targets in the metastatic setting are not currently available in NSCLC early stages, including KRAS.

KRAS is one of the isoforms, along with HRAS and NRAS, that belong to the RAS oncogene family. KRAS is the most frequently mutated and is found in approximately 15-20% of patients with NSCLC (27). The KRAS protein has a characteristic action, depending on a GTP-GDP mechanism, it oscillates between an active phase “ON” and an inactive phase “OFF”, allowing signal transduction to promote various cellular processes such as differentiation, growth, chemotaxis and apoptosis. This particular activation mechanism gathers the absence of well-defined hydrophobic pockets on the surface, picomolar affinity of GTP and GDP making mutations in KRAS difficult targets (28) as proved by the RR and PFS data in the metastatic setting with the selective inhibitors sotorasib and adagrasib (29, 30). Literature data have shown that KRAS mutations have a negative impact on prognosis (31), leading to resistance to most treatments, including checkpoint inhibitors, particularly when co-occurring with mutations like *STK11/LKB1* in metastatic setting (32). On the other hand, the available data on KRAS in the adjuvant setting come from the analysis of the LACE-bio trial and an old but large meta analysis, which showed no statistically significance in OS compared to wild-type and few data confirm no difference in OS between single hotspot mutation probably due to the small sample size (33, 34).

In our cohort of patients, 47 underwent radical surgery and were administered adjuvant CT with platinum-based therapy with gemcitabine. KRAS mutations exhibited a higher prevalence of PD-L1≥1% (56%; n=14), the most common mutations were G12C, G12V and G12A findings that align with the existing literature (11).

A subsequent analysis of survival data revealed a trend in favor of the WT population (mOS: 44.38 months 95% CI: 28.93-NA vs. 41.82 months 95% CI: 41.46-NA, *p=0.2*). However, when we analyzed the G12C subgroup compared to the other mutations, we did not find statistical significance in survival (mOS NR 95% CI: 28.93-NA vs. 34.78 months 95% CI: 21.73-NA, *p=0.25*).

This work aims to explore the potential of adding KRASi sequentially or concurrently to CT in two KRAS G12C mutated NSCLC cell lines. In this study, the CI with Gem+Carbo exerts the best viability reduction in SW1573-PR and H23-PR cell lines when KRASi are added sequentially or concurrently in 2D model and similar results were confirmed in spheroid models. The combination therapy was particularly effective in reducing the viability of SW1573-PR and H23-PR, outperforming other tested chemotherapeutic regimens such as Peme+Carbo and Pacli+Carbo. However, the efficacy of these agents is frequently compromised by the development of chemoresistance, a major obstacle in the successful management of the disease. In our cohort more than half of the patients (n=24) experienced a recurrence. RFS was shorter in KRAS mutated patients without a statistical significance (mRFS 25.84 months vs. 31.99 months; *p=0.23*) suggesting that the KRAS mutation may have a deleterious effect and that the tumor cells may be inherently resistant to adjuvant therapies. Gemcitabine resistance, in particular, poses a significant challenge, limiting the effectiveness of this widely used chemotherapeutic agent (35). Gemcitabine resistance in NSCLC involves multiple mechanisms as per autophagy suppression via impaired JNK-mediated Bcl-2 phosphorylation that limits autophagy-dependent cell death (36), enhancing activation of the PI3K/AKT/NF-κB pathway and reducing ROS-driven ERK signaling, with survival and proliferation boost as direct results (37). Another mechanism of resistance involves hypoxia-inducible factor-1α (HIF-1α) that upregulates ABCB6 expression, reprogramming heme metabolism to reduce reactive oxygen species (ROS) and conferring resistance (38). Similarly, targeting mTORC2, rather than mTORC1, sensitizes cells to gemcitabine by inducing apoptosis (39). The FOXO3-regulated TRIM22 axis promotes autophagy to protect cells from gemcitabine-induced apoptosis, further reducing drug sensitivity (40). Additionally, exosomal transfer of miR-222-3p drives gemcitabine resistance and malignancy by targeting SOCS3 (41). To better understand the mechanism behind gemcitabine chemoresistance in KRAS mutated cells we developed H23-GR and SW1573-GR cell lines, highlighting that chronic gemcitabine exposure induces significant transcriptional and cellular adaptations in NSCLC cells, promoting the development of resistance. Key resistance mechanisms include enhanced DNA damage repair, altered cell cycle dynamics, and an upregulation of transcriptional and translational machinery. The observed increase in the G0/G1 cell population in H23-GR cells, coupled with enrichment of pathways related to ribosome biogenesis, RNA processing, and chromatin remodeling, supports the hypothesis that resistant cells reprogram their metabolic and replicative machinery to adapt to therapeutic stress.

To determine whether gemcitabine resistance was reversible with the addition of a KRASi, we next replicated the analysis of cell viability on the two resistant cell lines H23-GR and SW1573-GR. Based on our previous results, we decided to treat both the 2D model and the spheroids directly with the CI+adagrasib combination, the most promising of the combination tested. Interestingly, both H23-GR and SW1573-GR exhibited similar sensibility to adagrasib used as a single agent or in combination with CI in 2D and 3D models. This finding suggests that KRASi may mitigate the adaptive resistance mechanisms in GR cells as already reported in preclinical PDAC models (42–44). Our 2D and 3D models demonstrated that the combination of CI+adagrasib was consistently more effective than CI or adagrasib alone in both PR and GR cells. The significant reduction in spheroid volume over extended treatment periods underscores the potential for combination regimens to achieve durable antitumor effects, even in the context of chemoresistance.

Importantly, while previous preclinical studies have investigated KRASi such as adagrasib and sotorasib in combination with CT (17, 19), our study provides several novel contributions. First, we employed both 2D and 3D spheroid models, the latter of which better mimics tumor architecture and drug penetration than conventional monolayer cultures (45). Second, we evaluated the effects of KRASi+CT combinations in chemoresistant cell lines—a clinically relevant context that remains underexplored in current literature. Third, we analyzed both concurrent and sequential treatment strategies, offering insights into potential therapeutic scheduling strategies that could optimize efficacy. These aspects collectively differentiate our work and underscore its translational relevance in a setting where therapeutic options for KRAS-mutant NSCLC remain limited in the adjuvant context.

## Conclusion

The results of this study highlight the potential impact of combination therapies in the early treatment of operated NSCLC, particularly for KRAS G12C tumors. The ability of KRASi to potentiate cytotoxic effects when combined with conventional chemotherapeutic agents provides a rationale for the integration of these agents into adjuvant treatment regimens. In addition, the efficacy observed in GR cell models suggests that KRASi may have a role to play in the treatment of chemoresistant disease. However, given the adverse event profile of individual agents, further *in vivo* safety evaluations are needed.

## Data Availability

The original contributions presented in the study are included in the article/**Supplementary Material**. Further inquiries can be directed to the corresponding author.

## Supplementary material

The Supplementary Material for this article can be found online at: https://www.frontiersin.org/articles/10.3389/fonc.2025.1654491/full#supplementary-material

Supplementary Figure 1 Dose-response curves (using GraphPad Prism) tested for chemotherapeutic agents and KRAS inhibitors in NSCLC cell lines with cell viability assay.

Supplementary Figure 2 Cell viability assay was performed in SW1573 or H23 cells treated for 72 hours with different combinations of chemotherapeutic agents and KRASi. Black bars indicated treatments at IC25 of the indicated drugs. Grey bars indicated treatments at IC50 of the indicated drugs. Data shown are mean ± SD from three independent experiments. *, P < 0.05; **, P < 0.01 refer to differences with respect to control (CTRL) as determined by Student t test; #, P < 0.05; ##, P < 0.01 refers to differences between the indicated samples as determined using one-way ANOVA. Δ indicates the percentage change in cell viability relative to the control (CTRL) or between the indicated samples.

Supplementary Figure 3
**(A)** Combined treatment of H23-PR and H23-GR NSCLC cells showing the effect of Gemcitabine, Carboplatin and Adagrasib or their combination on the phosphorylation of ERK1/2 and AKT. Vinculin was used as loading control. The graph below shows densitometric values of pERK1/2 and pAKT normalized for Vinculin content. Data shown are mean ± SD from two independent experiments. **(B, C)** Determination of spheroid volumes ± SD at different time points (3 and 7 days) normalized for the time point 0 (n = 3) comparing parental and GR SW1573 and H23 NSCLC cells. Cells were treated with different combinations of chemotherapeutic agents and KRASi Adagrasib at their IC50. Data were normalized to control and presented as mean ± SD from three independent experiments. *, P < 0.05; **, P < 0.01 refer to differences with respect to control (CTRL) as determined by Student t test.

## References

[1] PDQ® Adult Treatment Editorial Board. PDQ Non-Small Cell Lung Cancer Treatment. Bethesda, MD: National Cancer Institute. (2025) Available online at: https://www.cancer.gov/types/lung/hp/non-small-cell-lung-treatment-pdq (Accessed September 02, 2025)

[2] Duma N Santana-Davila R Molina JR. Non-small cell lung cancer: epidemiology, screening, diagnosis, and treatment. Mayo Clin Proc. (2019) 94:1623–40. doi: 10.1016/j.mayocp.2019.01.013, PMID: 31378236

[3] de Sousa VML Carvalho L. Heterogeneity in lung cancer. Pathobiology. (2018) 85:96–107. doi: 10.1159/000487440, PMID: 29635240

[4] Goldstraw P Chansky K Crowley J Rami-Porta R Asamura H Eberhardt WE. The IASLC lung cancer staging project: proposals for revision of the TNM stage groupings in the forthcoming (Eighth) edition of the TNM classification for lung cancer. J Thorac Oncol. (2016) 11:39–51. doi: 10.1016/j.jtho.2015.09.009, PMID: 26762738

[5] Bao R Chan P. Novel compounds in the treatment of lung cancer: current and developing therapeutic agents. J Exp Pharmacol. (2011) 3:21–34. doi: 10.2147/JEP.S7804, PMID: 27186107 PMC4863377

[6] Pignon JP Tribodet H Scagliotti GV Douillard JY Shepherd FA Stephens RJ. Lung adjuvant cisplatin evaluation: a pooled analysis by the LACE Collaborative Group. J Clin Oncol. (2008) 26:3552–9. doi: 10.1200/JCO.2007.13.9030, PMID: 18506026

[7] Felip E Altorki N Zhou C Vallières E Martínez-Martí A Rittmeyer A. Overall survival with adjuvant atezolizumab after chemotherapy in resected stage II-IIIA non-small-cell lung cancer (IMpower010): a randomised, multicentre, open-label, phase III trial. Ann Oncol. (2023) 34:907–19. doi: 10.1016/j.annonc.2023.07.001, PMID: 37467930

[8] Tsuboi M Herbst RS John T Kato T Majem M Grohé C. Overall survival with osimertinib in resected EGFR-mutated NSCLC. N Engl J Med. (2023) 389:137–47. doi: 10.1056/NEJMoa2304594, PMID: 37272535

[9] Wu YL Dziadziuszko R Ahn JS Barlesi F Nishio M Lee DH. Alectinib in resected ALK-positive non-small-cell lung cancer. N Engl J Med. (2024) 390:1265–76. doi: 10.1056/NEJMoa2310532, PMID: 38598794

[10] Fancelli S Petroni G Pillozzi S Antonuzzo L. Unconventional strategy could be the future: From target to KRAS broad range treatment. Heliyon. (2024) 10:e29739. doi: 10.1016/j.heliyon.2024.e29739, PMID: 38694108 PMC11061671

[11] Judd J Abdel Karim N Khan H Naqash AR Baca Y Xiu J. Characterization of KRAS mutation subtypes in non-small cell lung cancer. Mol Cancer Ther. (2021) 20:2577–84. doi: 10.1158/1535-7163.MCT-21-0201, PMID: 34518295 PMC9662933

[12] Riely GJ Marks J Pao W. KRAS mutations in non-small cell lung cancer. Proc Am Thorac Soc. (2009) 6:201–5. doi: 10.1513/pats.200809-107LC. Moore MJ, et al. gemcitabine: a review of the pharmacokinetics, pharmacodynamics, and clinical efficacy of gemcitabine. Clin Ther. 1997;19(5):1041-1053., PMID: 19349489

[13] Skoulidis F Byers LA Diao L Papadimitrakopoulou VA Tong P Izzo J. Co-occurring genomic alterations define major subsets of KRAS-mutant lung adenocarcinoma with distinct biology, immune profiles, and therapeutic vulnerabilities. Cancer Discov. (2015) 5:860–77. doi: 10.1158/2159-8290.CD-14-1236, PMID: 26069186 PMC4527963

[14] West HJ McCleland M Cappuzzo F Reck M Mok TS Jotte RM. Clinical efficacy of atezolizumab plus bevacizumab and chemotherapy in KRAS-mutated non-small cell lung cancer with STK11, KEAP1, or TP53 comutations: subgroup results from the phase III IMpower150 trial. J Immunother Cancer. (2022) 10:e003027. doi: 10.1136/jitc-2021-003027, PMID: 35190375 PMC8862451

[15] Fancelli S Caliman E Mazzoni F Paglialunga L Gatta Michelet MR Lavacchi D. KRAS G12 isoforms exert influence over up-front treatments: A retrospective, multicenter, Italian analysis of the impact of first-line immune checkpoint inhibitors in an NSCLC real-life population. Front Oncol. (2022) 12:968064. doi: 10.3389/fonc.2022.968064, PMID: 36452502 PMC9702560

[16] Skoulidis F Li BT Dy GK Price TJ Falchook GS Wolf J. sotorasib for lung cancers with KRAS p.G12C mutation. N Engl J Med. (2021) 384:2371–81. doi: 10.1056/NEJMoa2103695, PMID: 34096690 PMC9116274

[17] Canon J Rex K Saiki AY Mohr C Cooke K Bagal D. The clinical KRAS(G12C) inhibitor AMG 510 drives anti-tumour immunity. Nature. (2019) 575:217–23. doi: 10.1038/s41586-019-1694-1, PMID: 31666701

[18] Jänne PA Riely GJ Gadgeel SM Heist RS Ou SI Pacheco JM. Adagrasib in non-small-cell lung cancer harboring a *KRASG12C* mutation. N Engl J Med. (2022) 387:120–31. doi: 10.1056/NEJMoa2204619, PMID: 35658005

[19] Fell JB Fischer JP Baer BR Blake JF Bouhana K Briere DM. Identification of the clinical development candidate MRTX849, a covalent KRASG12C inhibitor for the treatment of cancer. J Med Chem. (2020) 63:6679–93. doi: 10.1021/acs.jmedchem.9b02052, PMID: 32250617

[20] Kim D Paggi JM Park C Bennett C Salzberg SL. Graph-based genome alignment and genotyping with HISAT2 and HISAT-genotype. Nat Biotechnol. (2019) 37:907–15. doi: 10.1038/s41587-019-0201-4, PMID: 31375807 PMC7605509

[21] Danecek P Bonfield JK Liddle J Marshall J Ohan V Pollard MO. Twelve years of SAMtools and BCFtools. Gigascience. (2021) 10:giab008. doi: 10.1093/gigascience/giab008, PMID: 33590861 PMC7931819

[22] Liao Y Smyth GK Shi W. featureCounts: an efficient general purpose program for assigning sequence reads to genomic features. Bioinformatics. (2014) 30:923–30. doi: 10.1093/bioinformatics/btt656, PMID: 24227677

[23] Love MI Huber W Anders S. Moderated estimation of fold change and dispersion for RNA-seq data with DESeq2. Genome Biol. (2014) 15:550. doi: 10.1186/s13059-014-0550-8, PMID: 25516281 PMC4302049

[24] Yu G Wang LG Han Y He QY. clusterProfiler: an R package for comparing biological themes among gene clusters. OMICS. (2012) 16:284–7. doi: 10.1089/omi.2011.0118, PMID: 22455463 PMC3339379

[25] Mohanty A Nam A Srivastava S Jones J Lomenick B Singhal SS. Acquired resistance to KRAS G12C small-molecule inhibitors via genetic/nongenetic mechanisms in lung cancer. Sci Adv. (2023) 9:eade3816. doi: 10.1126/sciadv.ade3816, PMID: 37831779 PMC10575592

[26] Lv D Hu Z Lu L Lu H Xu X. Three-dimensional cell culture: A powerful tool in tumor research and drug discovery. Oncol Lett. (2017) 14:6999–7010. doi: 10.3892/ol.2017.7134, PMID: 29344128 PMC5754907

[27] Chevallier M Borgeaud M Addeo A Friedlaender A. Oncogenic driver mutations in non-small cell lung cancer: Past, present and future. World J Clin Oncol. (2021) 12:217–37. doi: 10.5306/wjco.v12.i4.217, PMID: 33959476 PMC8085514

[28] Prior IA Lewis PD Mattos C. A comprehensive survey of Ras mutations in cancer. Cancer Res. (2012) 72:2457–67. doi: 10.1158/0008-5472.CAN-11-2612, PMID: 22589270 PMC3354961

[29] de Langen AJ Johnson ML Mazieres J Dingemans AC Mountzios G Pless M. Sotorasib versus docetaxel for previously treated non-small-cell lung cancer with KRASG12C mutation: a randomised, open-label, phase 3 trial. Lancet. (2023) 401:733–46. doi: 10.1016/S0140-6736(23)00221-0, PMID: 36764316

[30] Mok TSK Yao W Duruisseaux M Doucet L Azkárate Martínez A Gregorc V. KRYSTAL-12: Phase 3 study of adagrasib versus docetaxel in patients with previously treated advanced/metastatic non-small cell lung cancer (NSCLC) harboring a KRASG12C mutation. J Clin Oncol. (2024) 42:LBA8509. doi: 10.1200/JCO.2024.42.17_suppl.LBA8509

[31] Sun JM Hwang DW Ahn JS Ahn MJ Park K. Prognostic and predictive value of KRAS mutations in advanced non-small cell lung cancer. PloS One. (2013) 8:e64816. doi: 10.1371/journal.pone.0064816, PMID: 23724098 PMC3665805

[32] Skoulidis F Goldberg ME Greenawalt DM Hellmann MD Awad MM Gainor JF. STK11/LKB1 mutations and PD-1 inhibitor resistance in KRAS-mutant lung adenocarcinoma. Cancer Discov. (2018) 8:822–35. doi: 10.1158/2159-8290.CD-18-0099, PMID: 29773717 PMC6030433

[33] Shepherd FA Domerg C Hainaut P Jänne PA Pignon JP Graziano S. Pooled analysis of the prognostic and predictive effects of KRAS mutation status and KRAS mutation subtype in early-stage resected non-small-cell lung cancer in four trials of adjuvant chemotherapy. J Clin Oncol. (2013) 31:2173–81. doi: 10.1200/JCO.2012.48.1390, PMID: 23630215 PMC4881333

[34] Mascaux C Iannino N Martin B Paesmans M Berghmans T Dusart M. The role of RAS oncogene in survival of patients with lung cancer: a systematic review of the literature with meta-analysis. Br J Cancer. (2005) 92:131–9. doi: 10.1038/sj.bjc.6602258, PMID: 15597105 PMC2361730

[35] Jia Y Xie J. Promising molecular mechanisms responsible for gemcitabine resistance in cancer. Genes Dis. (2015) 2:299–306. doi: 10.1016/j.gendis.2015.07.003, PMID: 30258872 PMC6150077

[36] Chiu CH Ramesh S Liao PH Kuo WW Chen MC Kuo CH. Phosphorylation of Bcl-2 by JNK confers gemcitabine resistance in lung cancer cells by reducing autophagy-mediated cell death. Environ Toxicol. (2023) 38:2121–31. doi: 10.1002/tox.23836, PMID: 37219008

[37] Chiu CH Lin YJ Ramesh S Kuo WW Chen MC Kuo CH. Gemcitabine resistance in non-small cell lung cancer is mediated through activation of the PI3K/AKT/NF-κB pathway and suppression of ERK signaling by reactive oxygen species. J Biochem Mol Toxicol. (2023) 37:e23497. doi: 10.1002/jbt.23497, PMID: 37564025 PMC13546957

[38] Xiang L Wang Y Lan J Na F Wu S Gong Y. HIF-1-dependent heme synthesis promotes gemcitabine resistance in human non-small cell lung cancers via enhanced ABCB6 expression. Cell Mol Life Sci. (2022) 79:343. doi: 10.1007/s00018-022-04360-9, PMID: 35661930 PMC11072486

[39] Chawsheen MA Dash PR. mTOR modulates resistance to gemcitabine in lung cancer in an MTORC2 dependent mechanism. Cell Signal. (2021) 81:109934. doi: 10.1016/j.cellsig.2021.109934, PMID: 33545231

[40] Wang Y Liang HX Zhang CM Zou M Zou BB Wei W. FOXO3/TRIM22 axis abated the antitumor effect of gemcitabine in non-small cell lung cancer via autophagy induction. Transl Cancer Res. (2020) 9:937–48. doi: 10.21037/tcr.2019.12.33, PMID: 35117439 PMC8798778

[41] Wei F Ma C Zhou T Dong X Luo Q Geng L. Exosomes derived from gemcitabine-resistant cells transfer Malignant phenotypic traits via delivery of miRNA-222-3p. Mol Cancer. (2017) 16:132. doi: 10.1186/s12943-017-0694-8, PMID: 28743280 PMC5526308

[42] Lee JE Woo MG Jung KH Kang YW Shin SM Son MK. Combination therapy of the active KRAS-targeting antibody inRas37 and a PI3K inhibitor in pancreatic cancer. Biomol Ther (Seoul). (2022) 30:274–83. doi: 10.4062/biomolther.2021.145, PMID: 34663758 PMC9047487

[43] Lee JE Kang YW Jung KH Son MK Shin SM Kim JS. Intracellular KRAS-specific antibody enhances the anti-tumor efficacy of gemcitabine in pancreatic cancer by inducing endosomal escape. Cancer Lett. (2021) 507:97–111. doi: 10.1016/j.canlet.2021.03.015, PMID: 33744388

[44] Huang YK Cheng WC Kuo TT Yang JC Wu YC Wu HH. Inhibition of ADAM9 promotes the selective degradation of KRAS and sensitizes pancreatic cancers to chemotherapy. Nat Cancer. (2024) 5:400–19. doi: 10.1038/s43018-023-00720-x, PMID: 38267627

[45] Friedrich J Seidel C Ebner R Kunz-Schughart LA. Spheroid-based drug screen: considerations and practical approach. Nat Protoc. (2009) 4:309–24. doi: 10.1038/nprot.2008.226, PMID: 19214182

