# Supplementary figures and images for: KRAS G12C inhibition enhances efficacy to conventional chemotherapy in KRAS-mutant NSCLC
Source: Front Oncol. 2025 Sep 10;15:1654491. doi: 10.3389/fonc.2025.1654491 (PMC12457849; doi:10.3389/fonc.2025.1654491)

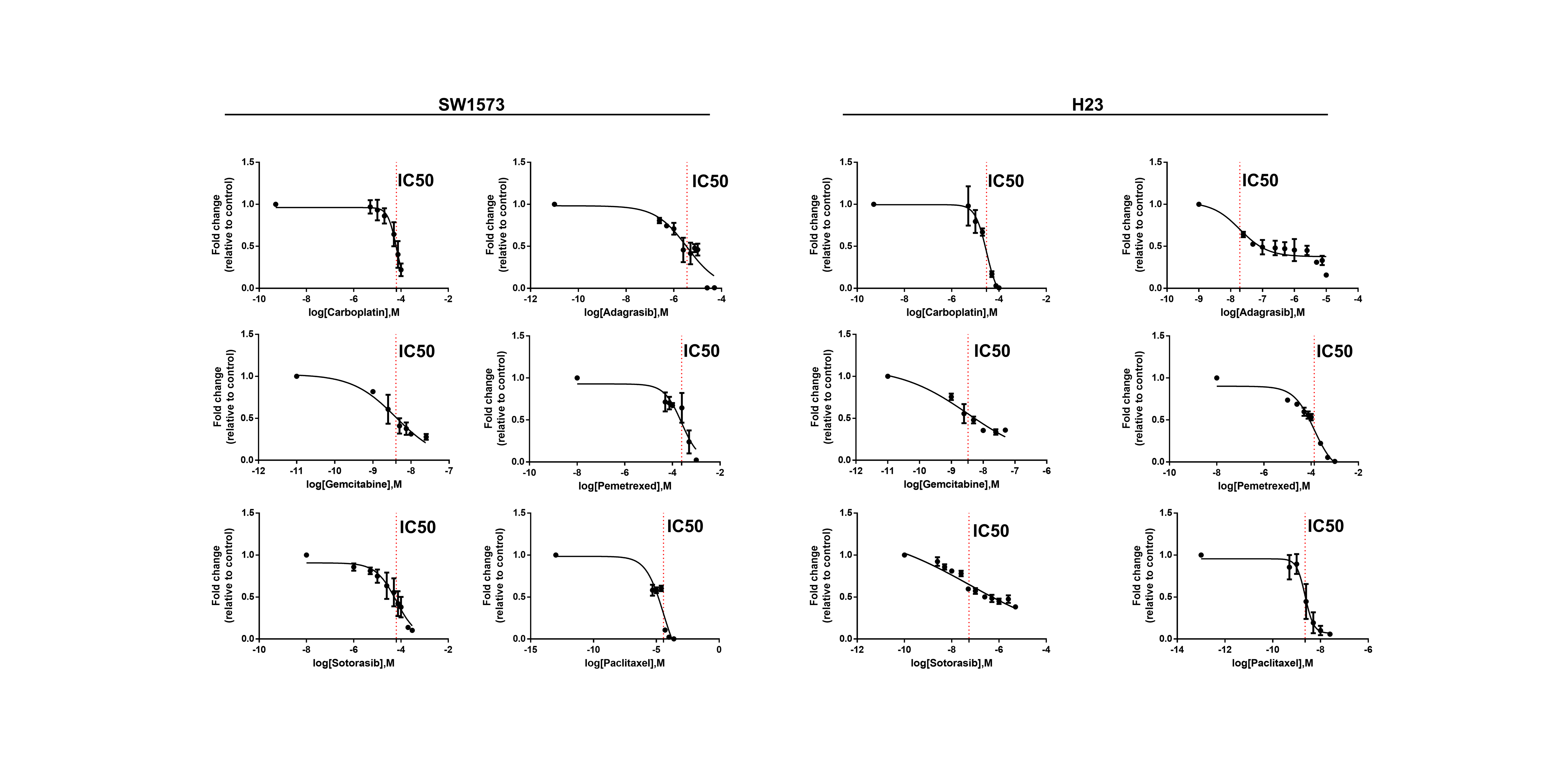

Supplement: Supplementary Figure 1 — Dose-response curves (using GraphPad Prism) tested for chemotherapeutic agents and KRAS inhibitors in NSCLC cell lines with cell viability assay. [file Image1.tif]

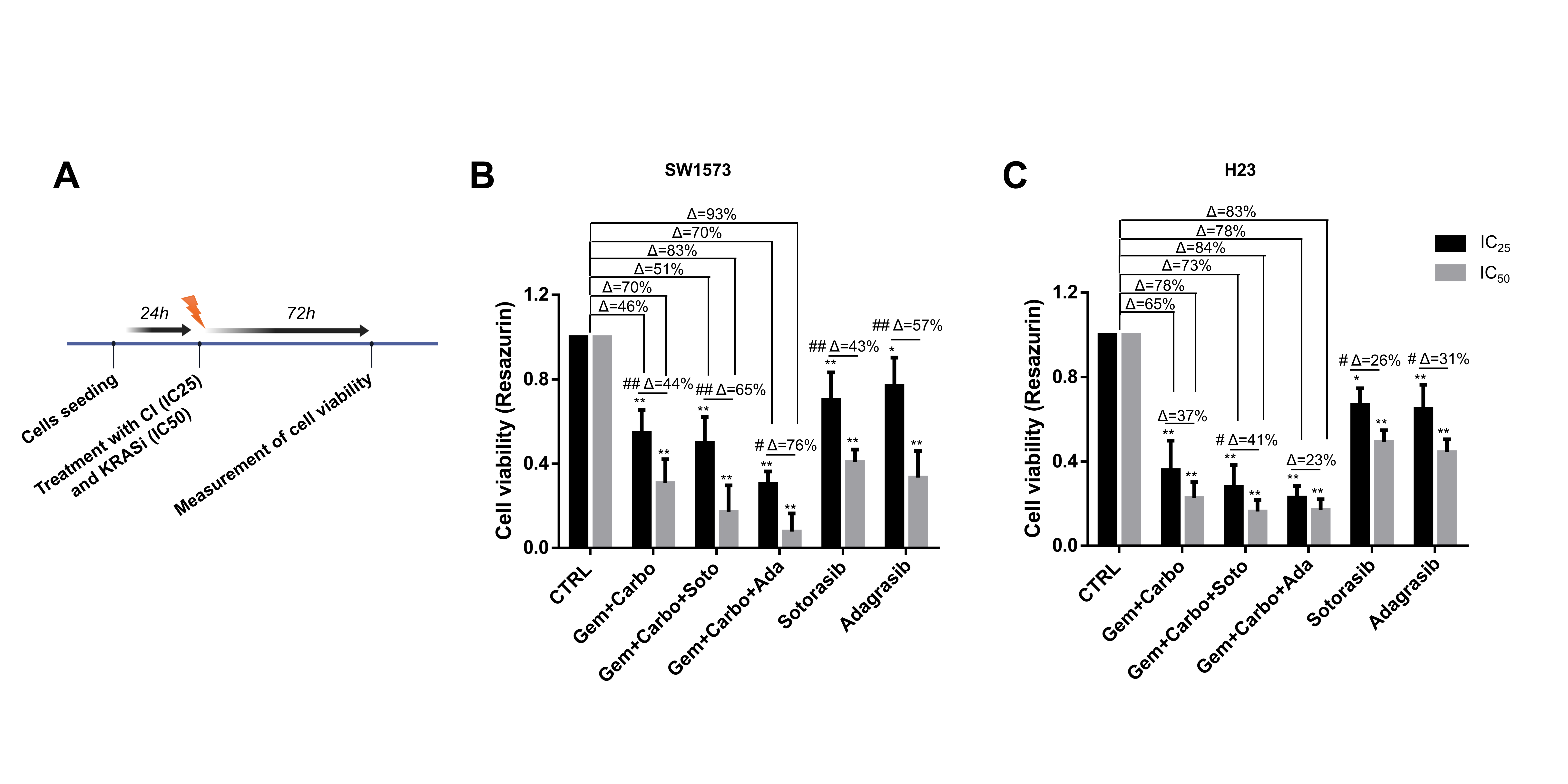

Supplement: Supplementary Figure 2 — Cell viability assay was performed in SW1573 or H23 cells treated for 72 hours with different combinations of chemotherapeutic agents and KRASi. Black bars indicated treatments at IC25 of the indicated drugs. Grey bars indicated treatments at IC50 of the indicated drugs. Data shown are mean ± SD from three independent experiments. *, P < 0.05; **, P < 0.01 refer to differences with respect to control (CTRL) as determined by Student t test; #, P < 0.05; ##, P < 0.01 refers to differences between the indicated samples as determined using one-way ANOVA. Δ indicates the percentage change in cell viability relative to the control (CTRL) or between the indicated samples. [file Image2.tif]

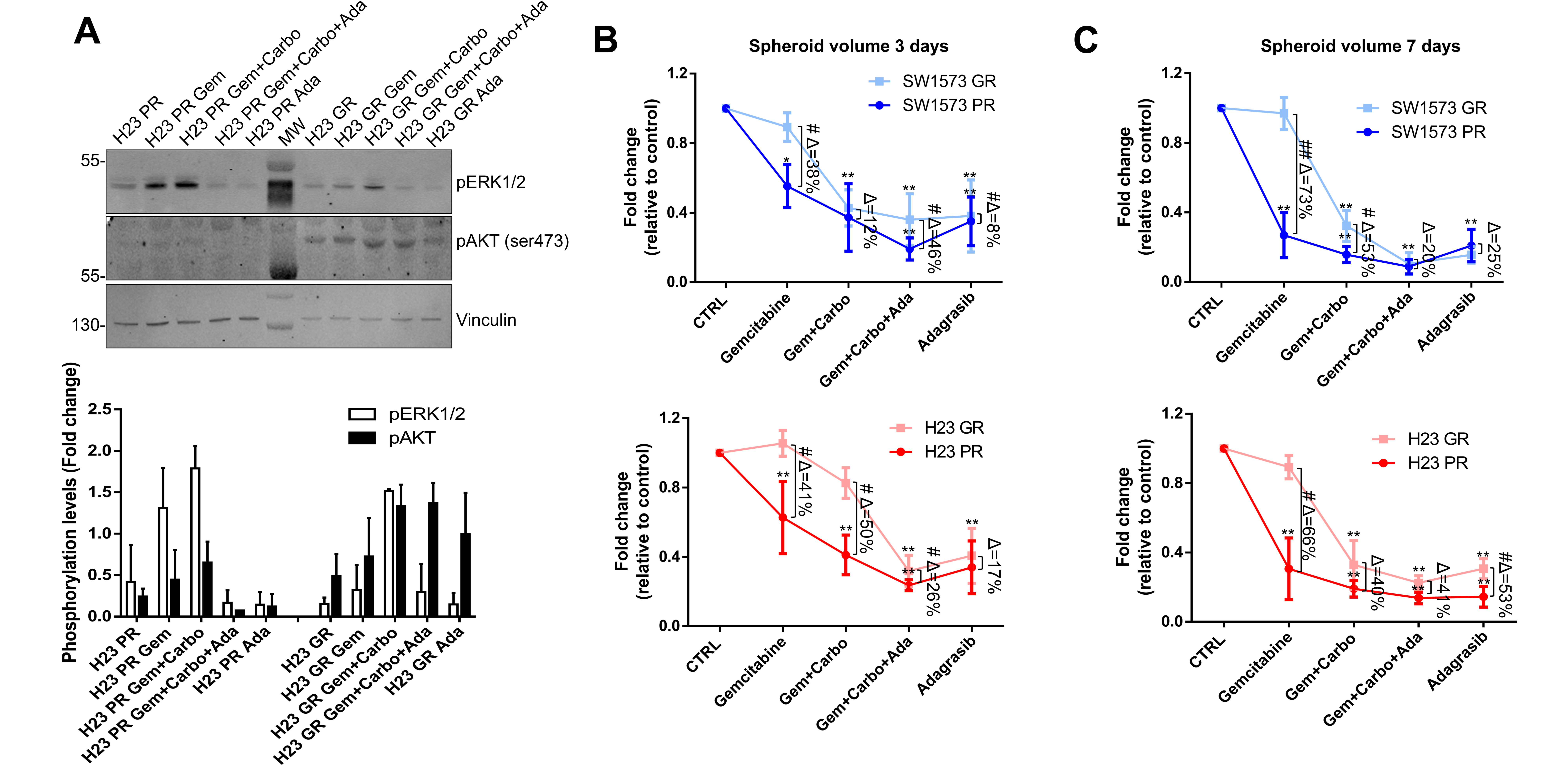

Supplement: Supplementary Figure 3 — (A) Combined treatment of H23-PR and H23-GR NSCLC cells showing the effect of Gemcitabine, Carboplatin and Adagrasib or their combination on the phosphorylation of ERK1/2 and AKT. Vinculin was used as loading control. The graph below shows densitometric values of pERK1/2 and pAKT normalized for Vinculin content. Data shown are mean ± SD from two independent experiments. (B, C) Determination of spheroid volumes ± SD at different time points (3 and 7 days) normalized for the time point 0 (n = 3) comparing parental and GR SW1573 and H23 NSCLC cells. Cells were treated with different combinations of chemotherapeutic agents and KRASi Adagrasib at their IC50. Data were normalized to control and presented as mean ± SD from three independent experiments. *, P < 0.05; **, P < 0.01 refer to differences with respect to control (CTRL) as determined by Student t test. [file Image3.tif]
